# Determination of prion proteins in the diagnosis of Creutzfeldt-Jakob disease using RT-QuIC: A case report from northeastern Colombia

**DOI:** 10.7705/biomedica.7352

**Published:** 2024-11-06

**Authors:** Jairo Lizarazo, Aixa Xiomara Vargas, Rafael Olarte, David Andrés Lizarazo

**Affiliations:** 1 Departamento de Medicina Interna, Universidad de Pamplona, Hospital Universitario Erasmo Meoz, Cúcuta, Colombia Hospital Universitario Erasmo Meoz Hospital Universitario Erasmo Meoz Cúcuta Cúcuta; 2 Unidad de Epidemiología, Hospital Universitario Erasmo Meoz, Cúcuta, Colombia Hospital Universitario Erasmo Meoz Hospital Universitario Erasmo Meoz Cúcuta Cúcuta; 3 Departmento de Radiología, Hospital Central de la Policía, Bogotá, D. C., Colombia Hospital Central de la Policía Hospital Central de la Policía Bogotá D. C Bogotá

**Keywords:** Prions, prion proteins, prion diseases, Creutzfeldt-Jakob syndrome, dementia, biomarkers, cerebrospinal fluid, priones, proteínas priónicas, enfermedades priónicas, síndrome de Creutzfeldt-Jakob, demencia, biomarcador, líquido cefalorraquídeo

## Abstract

Creutzfeldt-Jakob disease is a rare neurodegenerative disease caused by prions.

We present the case of a woman in the seventh decade of life with rapidly progressive dementia and myoclonus. Her brain magnetic resonance imaging revealed lesions in the basal nuclei, and the electroencephalogram showed periodic bilateral epileptiform discharges.

In the cerebrospinal fluid, the prion protein was detected using the real-time quaking-induced conversion test (RT-QuIC), and elevated levels of tau and 14-3-3 proteins. We emphasize the significance of determining the prion protein in the definitive diagnosis of this disease.

## Resumen

La enfermedad de Creutzfeldt-Jakob es una rara enfermedad neurodegenerativa causada por priones.

Se presenta el caso de una mujer en la séptima década de la vida con demencia rápidamente progresiva y mioclonías. La resonancia magnética cerebral mostró lesiones en los núcleos basales y el electroencefalograma evidenció descargas epileptiformes periódicas bilaterales.

En el líquido cefalorraquídeo, se detectó la proteína priónica mediante la prueba *Real-Time Quaking Induced Conversion* (RT-QuIC) y elevación de las proteínas tau y 14-3-3. Se enfatiza la importancia de identificar la proteína priónica en el diagnóstico definitivo de esta enfermedad.

Creutzfeldt-Jakob disease is a neurodegenerative disease caused by prions due to the accumulation of prion proteins (PrP^c^) with defects in folding (PrP^Sc^) in brain tissue, or a spontaneous mutation in the prion protein gene ^1^. There are three etiological types: sporadic, inherited, and acquired. Creutzfeldt-Jakob disease has a low global incidence with approximately 8590% of cases occurring sporadically ^2^.

Currently, the probable diagnosis of Creutzfeldt-Jakob disease combines clinical manifestations, abnormalities in electroencephalogram or brain magnetic resonance (MR) imaging, and biomarkers ^3^^,^^4^. Definitive diagnosis requires a pathological study of brain material to detect protease-resistant prion proteins (PrPres). In recent years, non-invasive diagnostic methods with high sensitivity and specificity have been developed, such as the real-time quaking-induced conversion (RT-QuIC) test, which detects misfolded prion proteins in cerebrospinal fluid and other tissues ^5^.

The RT-QuIC technique consists of a protein aggregation assay, where the self-propagating replication of abnormally folded prion protein (PrP^Sc^) is exploited to amplify minute amounts of PrP^Sen^ (recombinant sensitive protease polymerization) to a detectable level. A recombinant PrP^Sen^ solution is prepared in order to mimic the pathological process. This is distributed in plates that allow the ‘‘seeding’’ of a test sample and a control sample. If the sample contains the prion protein, it will cause the recombinant PrP^Sen^ to aggregate, as vigorous shaking causes fragmentation of the PrP^Sen^. Furthermore, PrP^Sen^ interacts with the dye thioflavin, which due to its fluorescent properties allows the response signal to be measured every 30 minutes ^6^. Despite its name, RT-QuIC is a long process that takes 90 hours. Currently, there is an adaptation of RT-QuIC called second generation that allows shortening the analysis time to about 30 hours ^7^.

We present a case of Creutzfeldt-Jakob disease, a sporadic variant in which the presence of prion protein in the cerebrospinal fluid was confirmed.

## Case presentation

A woman in her seventh decade of life consulted due to a month-long clinical picture that began with paranoid ideas and confabulation. This was followed by vertigo with balance disturbance, rapidly progressive cognitive deterioration, and a tendency toward mutism, in addition to dysphagia and loss of sphincter control. She had a history of high blood pressure, hypothyroidism, and hysterectomy plus salpingectomy for ovarian cancer. She took care of housework and was a vegetarian.

The physical examination on admission revealed an alert patient with bradylalia, bradyphrenia, poor speech, paralysis of vertical upward gaze, hypomimia, paratonic muscle rigidity, and myoclonus in all four extremities with generalized hyporeflexia (+/++++). The patient could not walk due to balance disturbance, had no sensory deficit, and had lost sphincter control. In the following weeks, greater neurological deterioration was evident; the patient became mute and had marked generalized hypertonia, multifocal myoclonus, and insomnia that required medication.

Laboratory tests revealed normal blood count, blood chemistry, serum electrolytes, and urinalysis but an elevated C-reactive protein level (28.70 mg/ dl); tests for HIV, syphilis, and hepatitis B-virus were negative.

**Figure 1:**
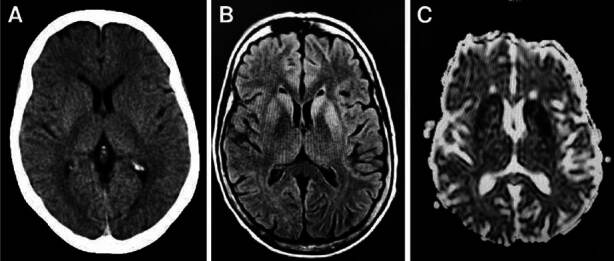
Sporadic Creutzfeldt-Jakob disease. A. Axial non-enhanced head computed tomography image shows no abnormal findings. B. Axial fluid-attenuated inversion recovery magnetic resonance image displays bilateral symmetric high signal intensity of the caudate heads and putamina. C. Axial Apparent Diffusion Coefficient (ADC) map reveals abnormally low diffusion in these regions.

The non-contrast head computed tomography (CT) was normal (figure 1A). A fluid-attenuated inversion recovery (FLAIR) magnetic resonance imaging (MR), without contrast agents, demonstrated a bilaterally symmetrical increase in signal intensity in the caudate nuclei and putamina and areas of decreased signal intensity in the apparent diffusion coefficient (ADC) map in the corresponding lesions seen on the FLAIR image (figures 1B and 1C).

**Figure 2:**
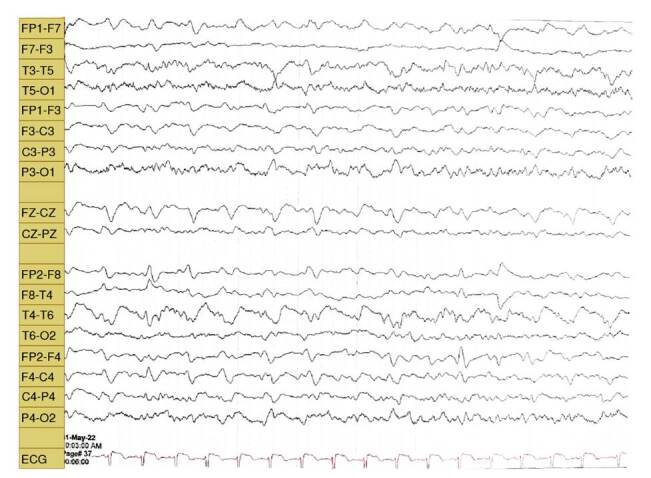
Electroencephalogram performed in patient’s awake state showing periodic, generalized, and synchronous sharp wave complexes.

The electroencephalogram showed complexes of periodic, generalized, synchronous sharp waves (figure 2).

The cerebrospinal fluid study revealed an opening pressure of 16 cm H_2_O and a normal cytochemical (0 cells, 0 red blood cells, proteins: 21.3 mg/ dl, glucose: 57 mg/dl) and microbiological examination (negative Gram stain and bacterial culture). The cerebrospinal fluid showed high levels of tau and 14-3-3 protein, and the second-generation RT-QuIC test was positive for prion proteins (table 1). These studies were performed at the National Prion Disease Pathology Surveillance Center in Cleveland, OH, United States of America.

**Table 1:** Results of prion proteins in the patient’s cerebrospinal fluid. Test were performed by The National Prion Disease Pathology Surveillance Center, Cleveland, OH, United States.

Test	Result	Normal reference range
RT-QuIC	Positive	Negative
T-tau protein (pg/ml)	> 20,000 pg/ml	0 - 1,149
14-3-3 protein (AU/ml)	> 160,000 AU/ml	< 30 - 1,999

AU: Arbitrary units

The patient’s neurological deterioration progressed, requiring gastrostomy and tracheostomy. Management was symptomatic with valproic acid and quetiapine. She was later discharged with home care. The patient was sent for palliative management at home after more than a month of hospitalization. The patient died 17 months after discharge. No autopsy was performed.

### Ethical aspects

Due to the patient’s mental incapacity, the informed consent was signed by the family members. They proved to be very interested in the publication of the case.

## Discussion

Sporadic Creutzfeldt-Jakob disease is a rare and fatal transmissible neurodegenerative entity with an annual incidence of 1.5 to 2 cases per million inhabitants ^2^. In Colombia, Escandón-Vargas *et al*. ^8^ reviewed 28 cases of this disease published in Colombian medical literature between 1973 and 2015, and reported a new case with positive 14-3-3 protein in the cerebrospinal fluid. Only half of these cases were notified to SIVIGILA (Colombian public health surveillance system) ^9^. In recent years, three new cases of Creutzfeldt-Jakob disease have been reported ^10^^-^^12^ with positive tau and 14-3-3 proteins in the cerebrospinal fluid; the last two cases also had positive prion proteins in the sample source ^11^^-^^12^. All Creutzfeldt-Jakob disease cases reported in Colombia have been sporadic ^8^^-^^12^.

The real incidence of prion diseases in Colombia is unknown but probably higher than described. Published cases, including our patient, comprise one case of scrapie and 33 cases of sporadic Creutzfeldt-Jakob disease; this country has no evidence of bovine spongiform encephalopathy ^8^. Most cases of sporadic Creutzfeldt-Jakob disease occur in people between 50 and 70 years old, which is congruent with the global literature reported data. This disease has been reported in different regions of the country with a random geographic distribution, without personal or family history suggesting iatrogenic causes or other neurodegenerative diseases. In general, the disease course was less than eight months ^8^.

Creutzfeldt-Jakob disease is caused by a proteinaceous infectious agent (prion - PrP^Sc^). When prions interact with the prion protein (Pr^PC^) -normally folded in the tissue-, it generates a conformational reorganization of the protein structure ^13^. The disease presents variable clinical presentations and pathophysiology. Its heterogeneity possibly lies in the aberrant structures the prion protein acquires. At least six phenotypes of sporadic Creutzfeldt- Jakob disease have been described based on clinical manifestations correlated with polymorphic variants and protein molecular weight after proteinase K-resistant proteolytic digestion (beta sheets). Each of them is likely caused by a peculiar prion protein strain ^13^^,^^14^.

Prion diseases are difficult to diagnose, as many other neurological disorders share the same symptoms, especially early in the disease. The clinical manifestations of Creutzfeldt-Jakob disease include multiple neurological and psychiatric abnormalities such as rapid progressive dementia, ataxia, and myoclonus. However, clinical findings vary substantially ^2^^,^^15^. Also, visual and cerebellar changes, pyramidal and extrapyramidal signs, and akinetic mutism often occur. Historically, the diagnosis of Creutzfeldt-Jakob disease has combined clinical manifestations, electroencephalogram, imaging studies, and cerebrospinal fluid biomarkers ^3^^,^^4^.

The electroencephalogram (EEG) is one of the key elements in the diagnosis of Creutzfeldt-Jakob disease ^16^. In the sporadic form, EEG shows changes that depend on the state of the disease, ranging from non-specific findings such as diffuse slowing and frontal intermittent rhythmic delta activity (FIRDA) in early stages to the typical periodic sharp wave complexes in the intermediate and late stages of the disease (figure 2). In the final stages, traces of non-reactive coma, or alpha coma can be found. Periodic sharp wave complexes can be lateralized (especially early in the disease) or generalized and appear in approximately 67% of patients with sporadic Creutzfeldt-Jakob disease, with a specificity of 91% and a positive predictive value of 95% ^16^.

Brain MRI is another pillar for Creutzfeldt-Jakob disease diagnosis ^17^. Diffusion-weighted imaging (DWI) and FLAIR imaging sequences have a sensitivity of 91% and a specificity of 95%. DWI images are more sensitive than FLAIR. Typically, they display focal hyperintense lesions that involve the cerebral cortex and deep gray matter (striatum and thalamus) ^17^. Apparent diffusion coefficient (ADC) hypointensity is often seen (associated with restricted diffusion) ^18^. DWI hyperintensity patterns differentiate Creutzfeldt-Jakob disease from other rapidly progressive dementias. Our patient only showed lesions in the striatum (caudate and putamen) (figure 1). MRI allows differential diagnosis with other metabolic, inflammatory, or infectious brain diseases ^19^.

Confirmation of the diagnosis requires a brain autopsy to identify the accumulation of prions, the only specific biomarker of the disease ^14^^,^^20^. Histological examination allows visualization of the classic characteristics of spongiosis, neuronal loss, prion protein deposition, and gliosis. Nevertheless, clinical and laboratory tests progressively introduced into clinical practice make it possible to reach a diagnosis based especially on biomarker determinations ^14^. Brain biopsy in patients with this suspected diagnosis is increasingly rare in practice due to the risks of disease transmission.

Tau and 14-3-3 proteins are surrogate cerebrospinal fluid biomarkers of Creutzfeldt-Jakob disease. These proteins are expressed in different organs, including the brain, and are located in the cytoplasm, plasma membranes, and organelles of cells. Tau protein is associated with microtubules and is expressed in neurons and glial cells. Clinically, it is often related to the study of Alzheimer’s and inflammatory and central nervous system neoplasias ^2^. There is controversy about the cut-off value for the diagnosis of Creutzfeldt-Jakob disease. However, very high values differentiate it from Alzheimer’s and other neurological diseases ^2^. The 14-3-3 protein has high specificity for discriminating sporadic Creutzfeldt-Jakob disease from other neurodegenerative conditions such as Alzheimer’s disease and Lewy body and frontotemporal dementia ^2^.

The RT-QuIC test was described for the first time in 2010 ^21^^,^^22^ and has become a specific biomarker applicable to cerebrospinal fluid, olfactory mucosa, urine, and skin, allowing the diagnosis of premortem disease given its high sensitivity and specificity. In a prospective study performed on cerebrospinal fluid samples, RT-QuIC showed a sensitivity of 96% and a specificity of 100%, significantly higher values compared to tau (85% sensitivity and 70% specificity) and 14-3-3 (84% sensitivity and 46% specificity) proteins. The RT-QuIC test achieved 100% sensitivity and specificity when researchers combined results from cerebrospinal fluid and olfactory mucosa samples ^5^. RT-QuIC has the advantage of being a non- invasive tool to confirm the diagnosis by prion protein detection in brain tissue ^2^. The technique is not affected by storing cerebrospinal fluid samples at room temperature or 4°C for up to eight days or by repeated freeze-thaw cycles. However, contamination with red blood cells (> 1,250 erythrocytes/ μΙ) does affect it since red cells inhibit the RT-QuIC response, resulting in a false negative ^5^. Another advantage is the absence of risks for patients or healthcare personnel ^3^.

Creutzfeldt-Jakob disease is an invariably fatal disease. A recent systematic review ^23^ of the different drugs used in patients with Creutzfeldt- Jakob disease showed that none of them were significantly effective in increasing patient survival. Flupirtine appears to have a beneficial effect in reducing cognitive decline in these patients, but this medicine is not available in our country. Nonetheless, additional studies are needed to establish therapeutic options to manage patients with Creutzfeldt-Jakob disease. The recognition of prion diseases constitutes a medical challenge due to their low incidence, different clinical manifestations, and difficulty in performing molecular tests in our country.

In this article, we present a case of Creutzfeldt-Jakob that, despite its rarity, is important to divulge due to the serious prognosis of the disease and its public health implications. The RT-QuIC test is an important advance in the diagnosis of this pathology. However, the high cost of the test and the need to send the sample abroad constitutes a great limitation.

## Conclusion

We present a typical clinical case of Creutzfeldt-Jakob disease, characterized by rapidly progressive dementia, myoclonus, and other neurological manifestations. It was supported by the electroencephalogram and brain magnetic resonance imaging findings and confirmed by the presence of tau and 14-3-3 proteins in the cerebrospinal fluid sample. The positive RT-QuIC test for prion proteins allowed us to improve the level of diagnostic certainty.
